# Evaluation of the precision and accuracy of augmented reality for pedicle screw placement in the cervical spine

**DOI:** 10.1016/j.xnsj.2025.100618

**Published:** 2025-05-29

**Authors:** Lisa M. Tamburini, Anthony Viola, Rohan R. Patel, Tomer Korabelnikov, Raghunandan Nayak, Justin S King, Scott Mallozzi, Isaac L. Moss, Hardeep Singh

**Affiliations:** Department of Orthopaedic Surgery, University of Connecticut, 120 Dowling Way, Farmington, CT 06032, United States

**Keywords:** Augmented reality, Cervical spine, Pedicle screws, Cadaveric, Precision, Accuracy

## Abstract

**Background:**

Augmented reality (AR) has gained popularity in spine surgery. Head mounted AR devices superimpose a 3D reconstructed model on patient anatomy which has been shown to assist with accurate placement of lumbar spine pedicle screws. We aimed to evaluate the accuracy and precision of AR in cervical spine pedicle screw placement.

**Methods:**

Seven fresh-frozen cadaveric C2-T1 specimens were used. Computed tomography (CT) scans were obtained and uploaded to the AR navigation system. Fiducial markers were utilized to ensure accurate registration. Bilateral C2-C7 pedicle screws were placed. Images containing planned trajectory with tap and navigated screw placement were captured. A post-navigation CT scan was obtained. Time from initial CT scan to navigation and total navigation time were recorded. Radiation dose information was obtained. Linear and angular differences between planned trajectory and navigated screw position as well as navigated screw position and actual screw position were measured on axial and sagittal images. Screw position was graded using the Gertzbein-Robbins classification.

**Results:**

82 pedicle screws were placed. The mean angular and linear deviation between the tap trajectory and navigated screw position were 2.63 ± 2.65° and 3.08 ± 2.32° and 1.11 ± 1.04 mm and 1.24 ± 0.84 mm in the axial and sagittal planes. The mean angular and linear deviation between navigated screw and actual screw were 3.68 ± 4.15° and 2.44 ± 2.17° and 1.51 ± 1.53 mm and 1.02 mm ± 0.88 in the axial and sagittal planes. 95% of screws were given a grade of A or B. Average time from CT scan to navigation was 139.4 seconds and average navigation time was 33 minutes and 46 seconds. Average radiation exposure time was 12.76 ± 1.57 seconds and the average dose-length product (DLP) was 551.15 ± 74.04 mGy-cm.

**Conclusions:**

AR can assist in accurate placement of pedicle screws in the cervical spine. Deviation from navigated screw position to actual screw position was within clinically acceptable range throughout the cervical spine.

## Introduction

The use of augmented reality (AR) in surgery has gained popularity in recent years. AR utilizes computed tomography (CT) images to create a computer-generated 3D image that is superimposed over actual patient anatomy displayed through a surgeon worn headset [1]. One of the most popular applications of AR in spine surgery is for pedicle screw instrumentation, due to proposed benefits of decreased radiation exposure and maintenance of procedure time with maintained accuracy of screw placement [1]. While the extent of these benefits have been explored in in the thoracic and lumbar spine, there has been limited application and few studies investigating AR navigated surgery in the cervical spine [1,2].

When placing pedicle screws in the cervical spine, there is greater inherent risk due to the anatomy of the cervical spine and close proximity of neural elements and vertebral artery. When planning for cervical spine surgery many surgeons obtain CT scans, MRIs, and angiograms to better understand patient specific anatomy [3,4]. In addition to preoperative imaging, intraoperative fluoroscopy is also utilized to assist with pedicle screw placement [4,5]. Even in the hands of experienced surgeons with good preoperative planning, the risk of cortical breach with free hand placement of pedicle screws in the cervical spine has been reported as being as high as 29% [3,[5], [6], [7]]. Given this risk, surgeons often opt for placement of lateral mass screws in the cervical spine despite studies showing biomechanical superiority of pedicle screws [8,9]. A few studies have shown acceptable rates of accurate pedicle screw placement with robotic assistance [10,11]. Additionally, a study by Ruiz- Cardozo et al. evaluated the use of AR for pedicle screw placement in the cervical spine and reported acceptable accuracy [12]. AR may provide an opportunity for safer, accurate placement of cervical pedicle screws capitalizing tools surgeons may already be experienced using in the lumbar and thoracic spine.

While the accuracy of AR assisted pedicle screw placement in the lumbar spine has been studied [2,[13], [14], [15], [16], [17]], few studies have evaluated accuracy in the cervical spine [12]. Due to advances in AR software and hardware required for navigation, this study evaluated the precision and accuracy of pedicle screw placement in the cervical spine with the assistance of an augmented reality navigation system.

## Methods

This study utilized 7 fresh-frozen C1 to T2 spine specimens (5M, 57.4 years) from MedCure (MedCure, Inc., Portland, OR). All specimens were thawed at room temperature for 24 hours prior to dissection and testing. They were kept moist with saline as needed throughout dissection and testing. The C1 vertebrae was removed from all specimens. All soft tissue was removed from the C2 vertebra, and any necessary soft tissue and ribs were removed from the T1 and T2 vertebrae to allow for proper stabilization. The C2 and T2 vertebrae were then potted in specially designed 3D-printed fixtures with Duz-All Polymethyl methacrylate (PMMA) all-purpose self-cure acrylic repair material (Keystone Industries, Gibbstown, NJ) that allowed for cranial and caudal stabilization.

### Pedicle screw placement

**Fig. 1:**
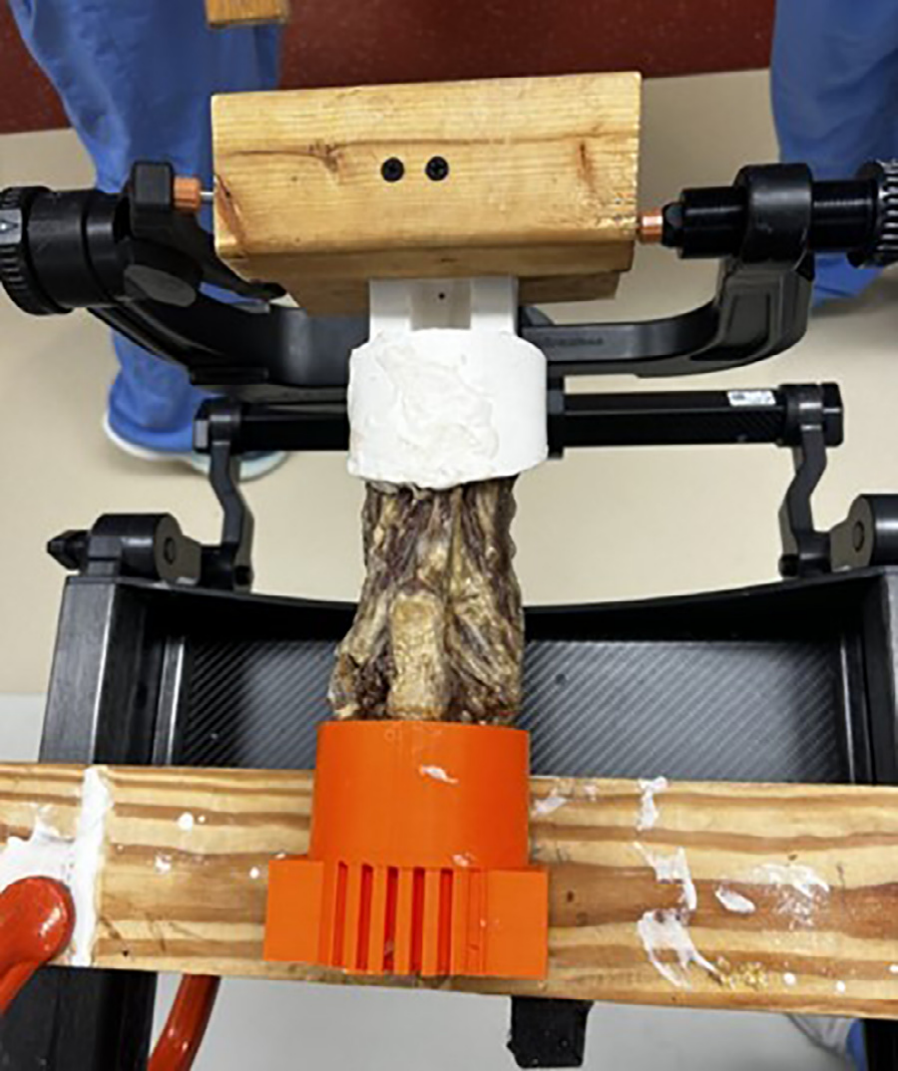
Cadaveric cervical spine was secured to operative table using Mayfield Skull Clamp cranially and C-clamps caudally.

The cadaveric spine was secured to the operating table in the prone position. The cranial pot was attached to a block of wood. A Mayfield Skull Clamp was tightened to the block of wood and then secured to the operative table. The caudal pot was attached to a block of wood which was secured to the operative table with C-clamps (Fig. 1). Four fiducial screws were placed into the lamina of randomly selected vertebrae. Each specimen had unique locations for fiducial screws to allow for identification of specimen, if needed. A navigation reference marker was attached to the spinous process of T1. A CT scan of the cadaver was obtained using the Airo TruCT © (Stryker, Leesburg, VA) and subsequently uploaded into the Xvision © navigation computer platform (Augmedics, Arlington Heights, IL). A 3D model was created using the system’s software. Accuracy of registration of the model relative to the tracker was confirmed by checking the location of the fiducial screws with a navigated probe. The navigated probe was placed on each screw, if the model showed the probe centrally located within the screw the model was considered accurate. If the probe was not on the screw in the model, re-registration would be required.

**Fig. 2:**
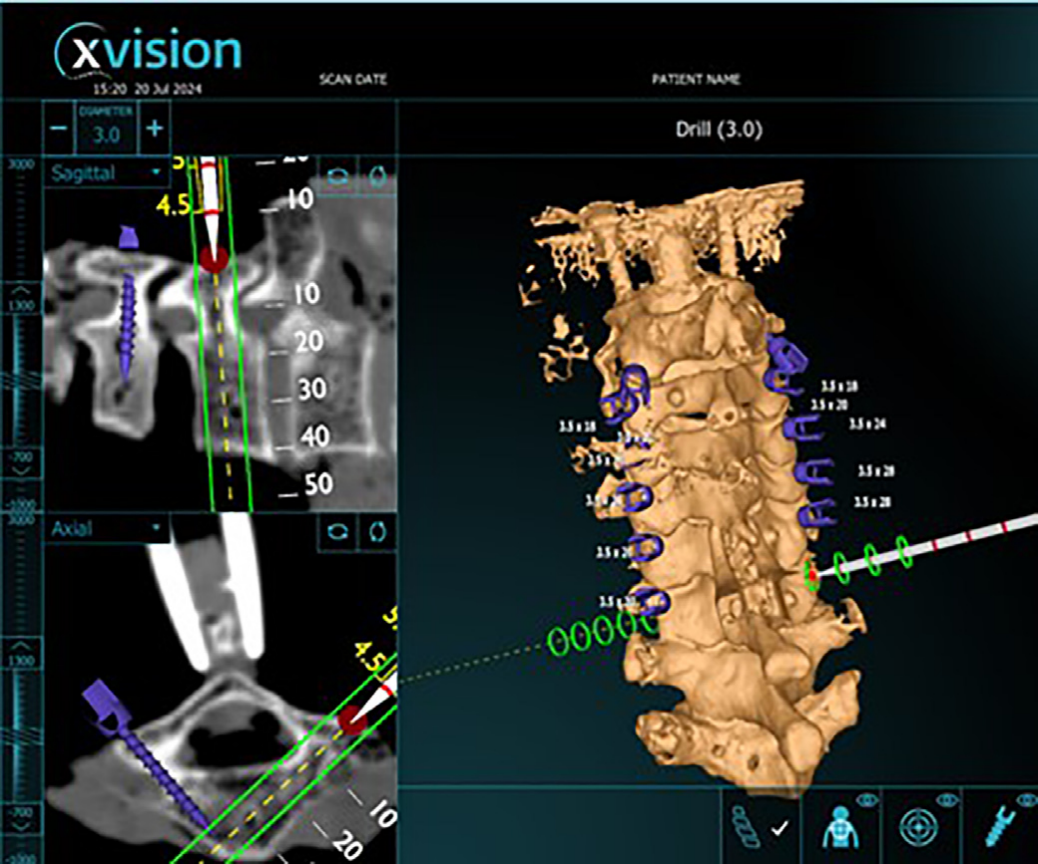
Screen shot showing initial trajectory on axial CT scan, sagittal CT scan and 3D model

**Fig. 3:**
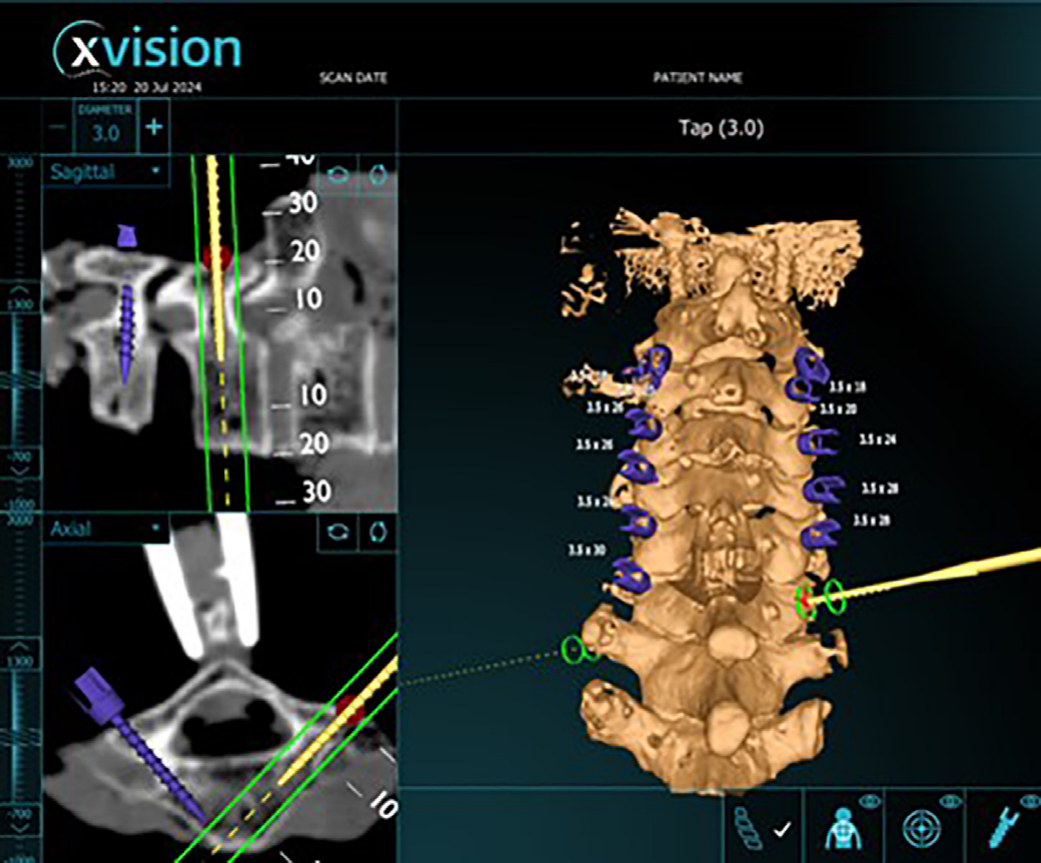
Screen shot showing tap on axial CT scan, sagittal CT scan, and 3D model.

**Fig. 4:**
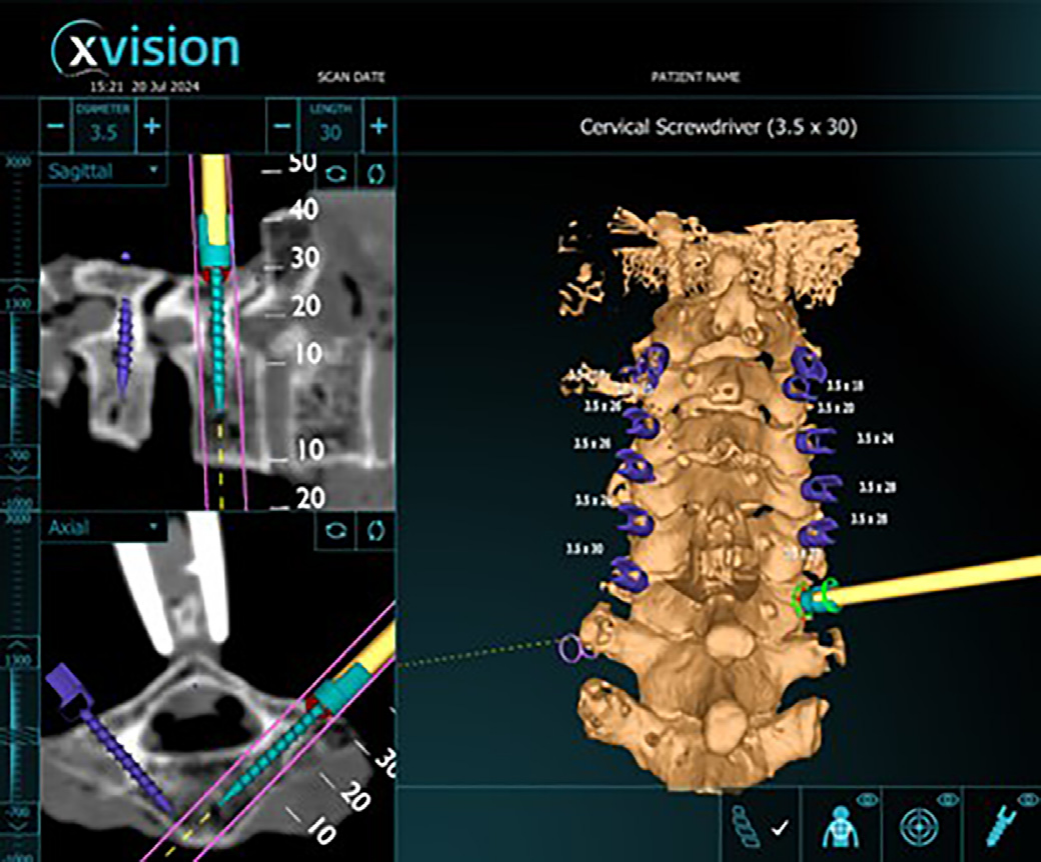
Screen shot showing final planned screw on axial CT scan, sagittal CT scan, and 3D model.

Pedicle screws were placed bilaterally from C2-C7 by a fellowship trained orthopedic spine surgeon and orthopedic surgery resident using X*vision* © augmented reality navigation system. The start point and initial trajectory was set with a navigated burr, which was used to make a pilot hole in cortical bone. An image highlighting the initial trajectory was captured (Fig. 2). Using this starting point, a tap was used to cannulate the trajectory for the screw. An image with the tap in place was captured (Fig. 3). Pedicle screws were then inserted in this trajectory. An image of the navigated screw position was captured (Fig. 4). 3.5 mm pedicle screws were used at every level. All captured images include axial and sagittal CT scan images and 3D model of the instrumented level with a projection of the associated instrumentation in place. If a breach was suspected during any step of instrumentation, the path was checked with a ball-tip probe and the trajectory was adjusted as needed. After all screws were implanted, a repeat CT scan was obtained.

Timing was recorded throughout. Duration of the initial CT scan, time from initial CT scan to navigation, and total navigation time was recorded for each specimen. Exposure time and Dose-Length Product (DLP) was recorded for initial CT scan. DLP was multiplied by 0.013 mSv/mGy/cm to get effective dose [18].

### Image processing

**Fig. 5:**
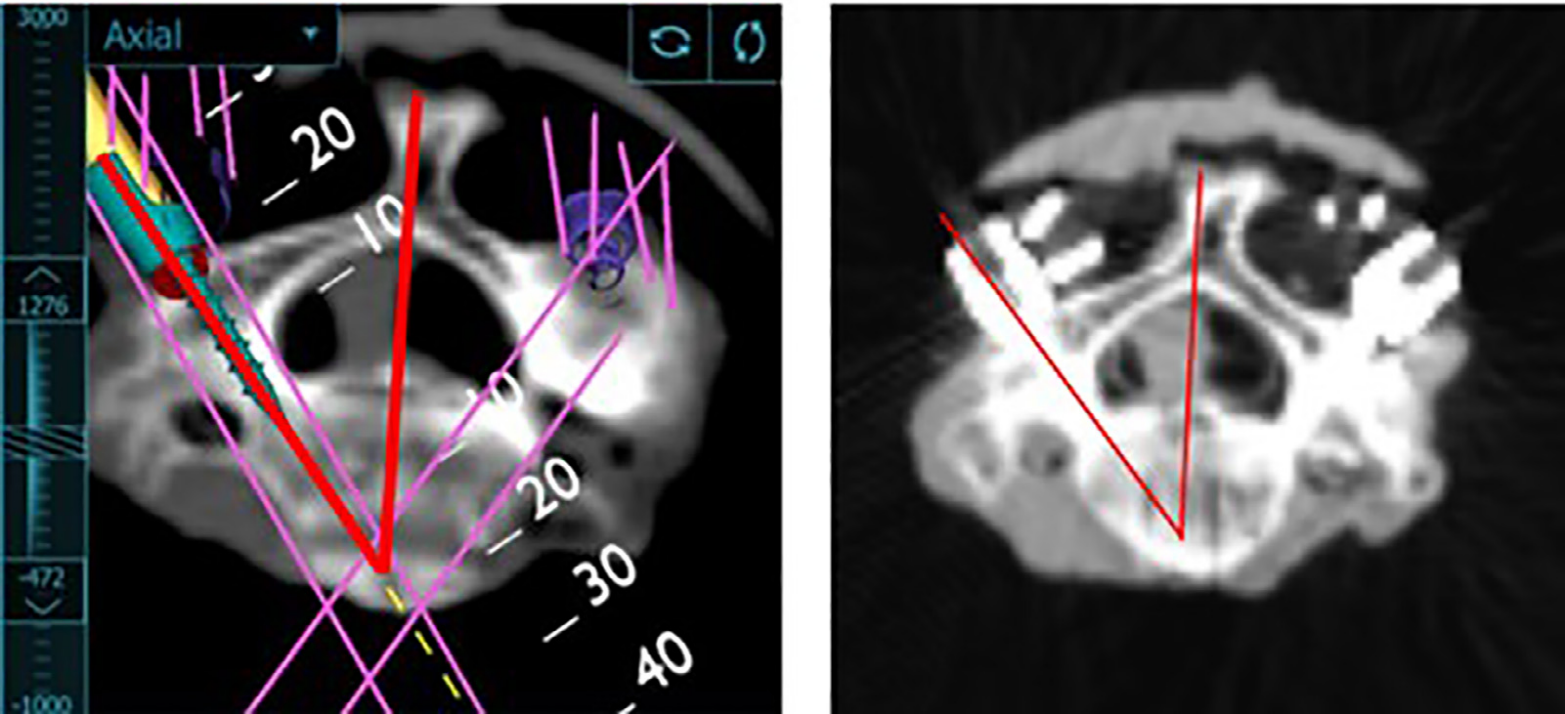
Axial image of final planned screw position from screen shot and axial CT scan image of final actual screw position from postoperative CT scan. Red lines indicate angles measured.

Axial and sagittal CT images of the tap, projected screw position, and actual screw position were uploaded to ImageJ (U.S. National Institutes of Health, Bethesda, MD). The angle of the tap, projected screw position, and actual screw position were measured using static anatomic landmarks such as the spinous process (Fig. 5). The angle of the tap was compared to the angle of the navigated screw position. Similarly, the angle of the projected screw position was compared to the actual screw position. Using these angles, the length of the screws, and trigonometric functions, the linear distance between the position of the tap and the tip of the final planned screw was calculated. The linear distance between the tip of the projected screw and the actual screw was also calculated. Mid-axial and mid-sagittal slices of the tap, projected screw, and actual screw were used. Measurements were completed by 3 independent raters.

**Fig. 6:**
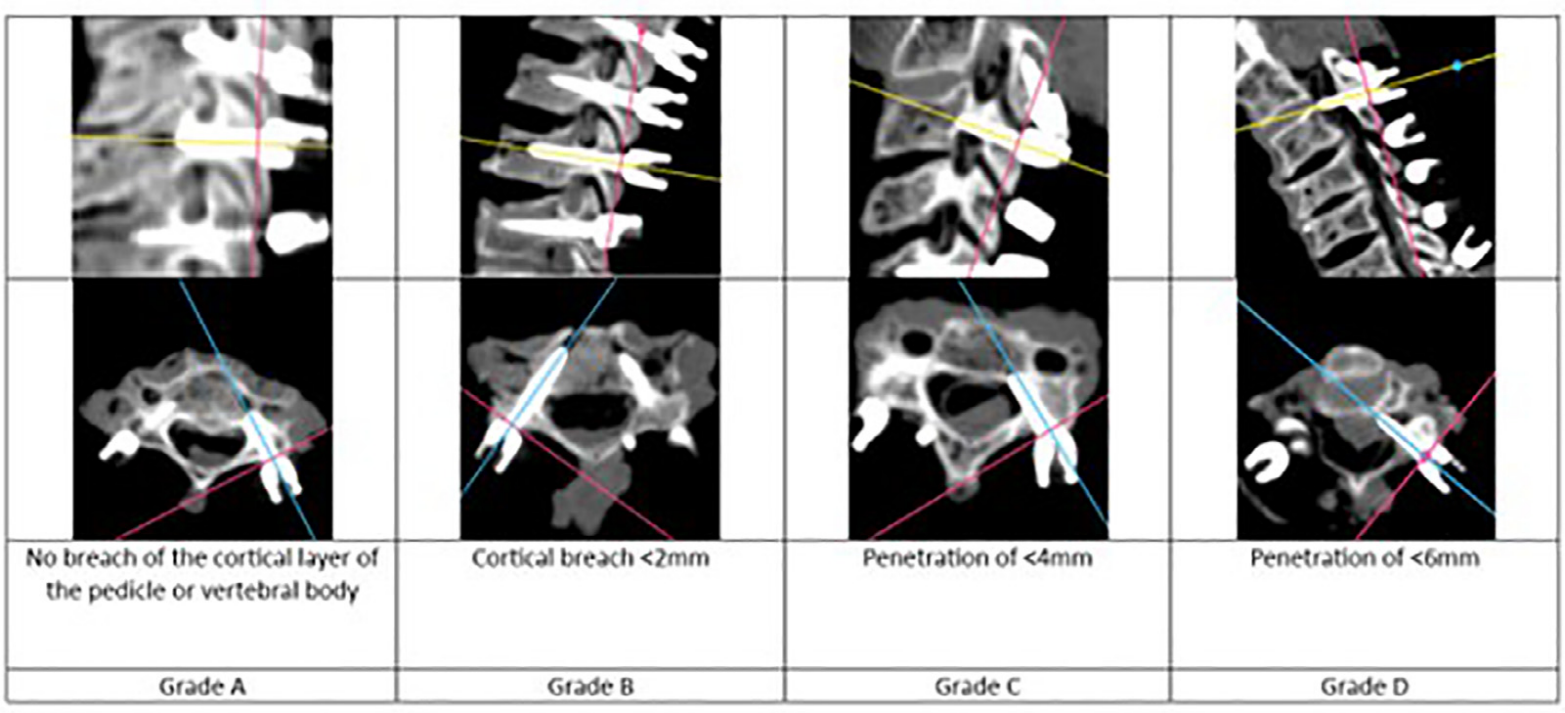
Representative axial and sagittal CT images of grades A-D Gertzbein- Robbins classification.

Accuracy of screw placement was grading using the Gertzbein-Robbins Classification, by assessing screw position on final axial and sagittal CT images [19]. Description and examples of A, B, C, and D grades are seen in Fig. 6. Each screw was graded by two independent graders.

Averages and standard deviations are presented for continuous variables. Percentages are presented for categorical variables. Interclass correlation coefficient (ICC) was calculated to ensure inter-rater reliability. Subgroup analysis was performed to compare upper cervical spine (C2-C4) to lower cervical spine (C5–C7).

## Results

### Accuracy and precision

Using fiducial screws, all models were determined to be accurate, and re-registration was not required for any specimen. Pedicle screw precision was measured twice. C2 for specimen 1 was not included in precision or accuracy measurements as the potting material precluded appropriate access for placement of pedicle screws.

First, we measured the angular and linear difference between the tap trajectory and the projected screw position. These were reported as absolute values. Average angular difference was 2.63 ± 2.65° and 3.08 ± 2.32° in the axial and sagittal planes, respectively. Average linear difference was 1.11 ± 1.04 mm and 1.24 ± 0.84 mm in the axial and sagittal planes, respectively. We further analyzed the precision of screw placement in the upper cervical spine (C2–C4) compared to the lower cervical spine (C5–C7). The average angular difference in the upper cervical spine was 3.00° and 3.87° in the axial and sagittal planes, respectively. The average angular difference in the lower cervical spine was 2.26° and 2.29° in the axial and sagittal planes, respectively. The average linear difference in the upper cervical spine was 1.10 mm and 1.37 mm in the axial and sagittal planes, respectively. The average linear difference in the lower cervical spine was 1.10 mm and 1.12 mm in the axial and sagittal planes, respectively. Angular and linear differences between tap trajectory and navigated screw for each level are presented in Table 1. Interrater reliability was ensured with an ICC of 0.993 for the tap trajectory vs navigated screw measurements.

**Table 1:** Difference in angular and linear distance between tap trajectory and navigated screw

		C2	C3	C4	C5	C6	C7	All levels
Angular difference (degrees)	Axial	2.61 ± 2.49	3.22 ± 4.83	3.16 ± 2.14	2.00 ± 1.53	2.20 ± 1.54	2.57 ± 2.09	2.63 ± 2.65
	Sagittal	4.8 ± 2.71	4.14 ± 2.79	2.67 ± 2.15	2.34 ± 1.88	2.04 ± 1.52	2.48 ± 1.39	3.08 ± 2.32
Linear difference (mm)	Axial	0.82 ± 0.81	1.15 ± 1.67	1.34 ± 0.97	0.94 ± 0.71	1.02 ± 0.70	1.35 ± 1.09	1.11 ± 1.04
	Sagittal	1.54 ± 0.90	1.47 ± 0.96	1.09 ± 0.84	1.10 ± 0.88	0.96 ± 0.73	1.30 ± 0.73	1.24 ± 0.84

We then measured angular and linear differences between the projected screw and the actual screw position. Average angular difference was 3.68 ± 4.15° and 2.44 ± 2.17° in the axial and sagittal planes, respectively. Average linear difference was 1.51 ± 1.53 mm and 1.02 ± 0.88 mm in the axial and sagittal planes, respectively. We further analyzed the precision of screw placement in the upper cervical spine compared to the lower cervical spine. The average angular difference in the upper cervical spine was 4.56° and 2.96° in the axial and sagittal planes, respectively. The average angular difference in the lower cervical spine was 2.88° and 1.92° in the axial and sagittal planes, respectively. The average linear difference in the upper cervical spine was 1.62 mm and 1.10 mm in the axial and sagittal planes, respectively. The average linear difference in the lower cervical spine was 1.41 mm and 0.95 mm in the axial and sagittal planes, respectively. Angular and linear differences between projected and actual screw for each level are presented in Table 2. Interrater reliability was ensured with an ICC of 0.962 for the projected vs. actual screw measurements.

**Table 2:** Difference in angular and linear distance between projected screw and actual screw

		C2	C3	C4	C5	C6	C7	All levels
Angular difference (degrees)	Axial	5.54 ± 5.78	4.76 ± 6.63	3.4 ± 2.88	3.51 ± 3.04	2.75 ± 2.26	2.39 ± 2.21	3.68 ± 4.15
	Sagittal	3.99 ± 2.67	2.47 ± 1.61	2.41 ± 2.66	1.56 ± 1.05	2.19 ± 2.53	2.02 ± 1.46	2.44 ± 2.17
Linear difference (mm)	Axial	1.74 ± 1.81	1.67 ± 2.30	1.47 ± 1.28	1.66 ± 1.45	1.31 ± 1.11	1.25 ± 1.16	1.51 ± 1.53
	Sagittal	1.36 ± 0.80	0.89 ± 0.57	1.06 ± 1.20	0.74 ± 0.51	1.04 ± 1.23	1.06 ± 0.76	1.02 ± 0.88

Using the Gertzbein-Robbins classification, 75.0% of screws were graded as an A, 20.1% were a B, 3.0% were a C, and 1.8% were a D. We then looked independently at the upper and lower cervical spine grades. 73.8%, 20.0%, 3.8%, and 2.5% of screws in the upper cervical spine were graded as an A, B, C, and D, respectively. 76.2%, 20.2%, 2.4%, and 1.2% of screws in the lower cervical spine were graded as an A, B, C, and D, respectively. Interrater agreement for grading was 92.8%.

### Time and radiation

Average initial CT scan time was 119 seconds (range: 85–158 seconds). Average time from CT scan to navigation was 139.4 seconds (range: 124–155 seconds). Average Total navigation time is 33 minutes and 46 seconds (range: 26 minutes and 4 seconds- 46 minutes and 44 seconds.

Average radiation exposure time for initial CT scans was 12.76 ± 1.57 seconds. Average DLP for initial CT scans was 551.15 ± 74.04 mGy-cm. This corresponds to an effective dose of 7.16 ± 0.96 mSv. Exposure time, dose-length product, and effective dose for initial CT scan for each specimen is presented in Table 3. The operative team had no exposure to radiation as they were able to step out of the room during CT scan.

**Table 3:** Radiation data per specimen

	Exposure time (s)	Dose-length product (DLP) (mGy-cm)	Effective dose (mSv)
Specimen 1	11.52	492.82	6.41
Specimen 2	13.44	583.13	7.58
Specimen 3	14.16	617.00	8.02
Specimen 4	13.76	598.19	7.78
Specimen 5	11.08	472.12	6.14
Specimen 6	10.80	458.95	5.97
Specimen 7	14.56	635.82	8.27
Average	12.76 ± 1.57	551.15 ± 74.04	7.16 ± 0.96

## Discussion

The use of AR in spine surgery heavily depends on the hardware-software-user interface’s ability to create an accurate model and track instruments relative to this model. In our study we used fiducial screws placed in the lamina to determine accuracy of the model. The location of all fiducial screws demonstrated accuracy of the model and no re-imaging or re-registration of any specimen required. Many studies have evaluated the ability of different software systems to create acceptable spine models to allow for safe placement of pedicle screws for robust fixation [[20], [21], [22]]. A study by Winkler et al., evaluated the accuracy of a CT scan created model and found their model to be within 2.1 mm and determined this to be acceptable surgical accuracy [22]. This same study found the highest accuracy was at the medial and lateral walls of the pedicle [22].

Once an accurate model has been created, this model can be used to place pedicle screws. The use of AR for pedicle screw placement in the thoracic and lumbar spine has been studied in the lab setting as well as in the clinical setting [2,[13], [14], [15], [16], [17]]. It has been shown to improve accuracy of pedicle screw placement in the lumbar spine. Yahanda et al. used the Gertzbein-Robins classification and found 100% accuracy of 63 screws in the thoracic and lumbar spine of 9 patients [14]. Screws graded as A and B were classified as accurately placed. In our study, 95% of screws (94% in the upper cervical spine and 96% in the lower cervical spine) were graded as accurately placed. This accuracy is similar to the accuracy seen in a study by Elmi-Terander et al who compared accuracy of AR placed screws to free hand screws and found 93.9% accuracy of AR placed screws and 89.6% accuracy of free hand screws [17]. Ruiz-Cardozo et al. also evaluated the use of AR for placement of pedicle screws in the cervical spine and found acceptable accuracy [12]. Our cadaveric study found similar results and adds to the growing body of literature of the use of AR for placement of pedicle screws.

Studies have also reported on linear and angular deviation of AR assisted pedicle screw placement in the lumbar spine. Youssef et al reported a mean linear deviation from 1.3 to 5.99 mm and a mean angular deviation of 1.6° to 5.88° [23]. In our study we were able to more precisely place screws utilizing AR with an average linear deviation of 1.51 mm in the axial plane and 1.02 mm in the sagittal plane and an average angular deviation of 3.68° in the axial plane and 2.44° in the sagittal plane. Additionally, we found greater accuracy in the lower cervical spine when compared to the upper cervical spine. All spines were instrumented caudally from C2 to C7. This supports the notion that despite the mobility in the cervical spine the 3D model remains accurate over time and useful for navigation. The difference seen between screws placed in the upper cervical spine and the lower cervical spine can be attributed to multiple factors. Vertebrae closer to the reference marker have a higher accuracy than vertebrae farther from the marker [11]. Additionally, non-clinical factors including the positioning of the cervical spine which may not be representative of in vivo positioning may contribute to the difference observed.

As the negative side effects of excessive radiation has been repeatedly demonstrated, multiple strategies should be implemented to mitigate its deleterious effects [24]. The use of AR can assist with decreasing this radiation exposure. Surgeons and surgical staff are able to step out of the room or move far away from the CT scanner during the initial CT scan which protects them from exposure. We report an average effective dose of 7.16 ± 0.96 mSv which is similar to the effective dose of a routine lumbar CT scan and an average DLP of 551.15 mGy-cm which is within the diagnostic reference level reported for cervical spine CT scans [25,26]. Implementation of low dose CT protocols can further reduce this effective dose [21]. This reduction in radiation exposure is important in light of the current increased risk of cancer and cataracts seen in orthopedic surgeons [27,28].

When implementing new technology there is typically a learning curve for both attending surgeons and trainees which can lead to increased OR time. Studies have shown that surgeons can implement AR without a lengthy learning curve and the accuracy in screw placement is seen when screws are placed by attending surgeons as well as by trainees [13,29]. In our study, pre-procedure CT scans did not add a significant amount of time to the overall procedure time, adding less than 3 minutes of time. Although during surgical conditions there are additional steps, including positioning and draping the patient for the scan which take time, our study shows the actual CT scan does not add a significant amount of time to the procedure. Mean navigation time for the placement of 12 screws was 33 minutes and 46 seconds. Additionally, we saw an improvement in total navigation time from specimen 1 to specimen 7, indicating a learning curve which can continue to improve with use of the AR navigation system.

The biomechanical strength of pedicle screws in the cervical spine has been compared to the strength of lateral mass screws. It has been found that pedicle screws have a higher pull-out strength and a lower rate of loosening and therefore are the better choice biomechanically [8,9]. Despite this, many surgeons prefer lateral mass screws in the cervical spine due to the risk of neural element and vertebral artery injury. The use of AR for accurate pedicle screw placement can help to mitigate some risk while gaining the biomechanical benefit of pedicle screw use.

While this study shows very promising results in support of the use of AR for placement of pedicle screws in the cervical spine, it is not without limitations. The cadaveric nature of this study provides promising baseline efficacy data encouraging further work evaluating its utility. The model used in our current study is a spine only cadaveric model with all soft tissues removed. This model required us to use materials to stabilize the spine to position it on the table which may artificially make navigation more or less difficult. Further studies should be performed on cadaveric specimen that include the head, neck, and torso which may be more representative of surgical conditions. Despite this, our study provides encouraging data in support of AR utilization in the cervical spine. Given the small pedicle diameters encountered in the cervical spine, a few millimeters difference in the can result in violation of the spinal canal or vertebral canal. However, the calculated linear and angular differences calculated in this study can be deemed acceptable as the majority of our screws demonstrated Grade A placement on the Gertzbein-Robbins classification system.

Our study adds to the growing body of literature regarding the use of AR in surgery. While the use of AR continues to become more prevalent continued evaluation of its efficacy is necessary while also considered limitations of AR including cost and the potential for increased dependency on technology.

## Conclusion

In this cadaveric study, we demonstrated acceptable precision and accuracy of pedicle screw placement in the cervical spine using AR navigation. Further, we demonstrated better precision and accuracy in the lower cervical spine when compared to the upper cervical spine. This precision and accuracy is seen while maintaining acceptable procedure times and radiation exposure equivalent to that of a lumbar spine CT. Together, these findings support further investigation of the use of AR for pedicle screw placement in the cervical spine.

## Declarations of competing interest

One or more of the authors declare financial or professional relationships on ICMJE-NASSJ disclosure forms.
